# Bis[μ_3_-2-(pyridin-3-yl)acetato-κ^3^ *O*:*O*:*O*′]bis­[μ_2_-2-(pyridin-3-yl)acetato-κ^2^ *O*:*O*′]bis­[chlorido­(1,10-phenanthroline-κ^2^ *N*,*N*′)dysprosium(III)]

**DOI:** 10.1107/S2414314622002310

**Published:** 2022-03-31

**Authors:** Yan Lin, Jian-Ping Yu

**Affiliations:** a College of Chemistry and Chemical Engineering, Gannan Normal University, Ganzhou, 341000, People’s Republic of China; Katholieke Universiteit Leuven, Belgium

**Keywords:** crystal structure

## Abstract

The title Dy^III^ complex, [Dy_2_(C_7_H_6_NO_2_)_4_Cl_2_(C_12_H_8_N_2_)_2_] or [Dy_2_(*μ*_3_-PAA)_4_(Cl)_2_(phen)_2_] (PAA = 3-pyridyl­acetate, phen = 1,10-phenanthroline), obtained by reaction of Dy(ClO_4_)_3_, 3-pyridyl­acetic acid ligands and 1,10-phenanthroline, exhibits a dinuclear structure. Adjacent binuclear dimers are further connected *via* face-to-face *π*–*π* stacking inter­actions resulting in supra­molecular chains along the *c*-axis direction.

## Abstract 2

A novel Dy^III^ complex based on 3-pyridyl­acetic acid and 1,10-phenanthroline ligands shows a dinuclear structure.

## Graphical abstract

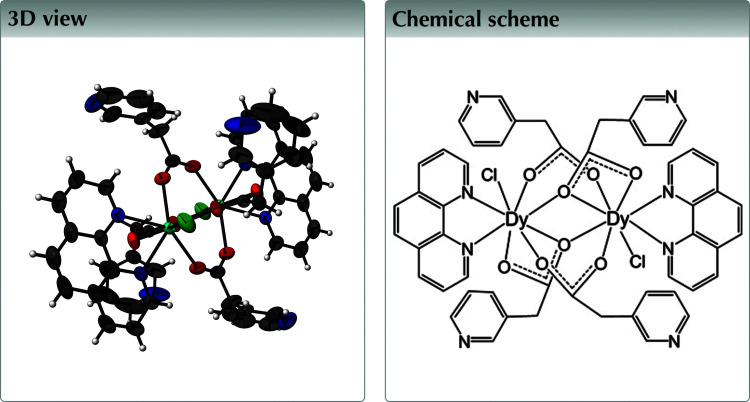

## Structure description

Coordination complexes composed of metal cations and organic ligands have received much attention because of their diverse structures and intriguing properties such as photoluminescence, magnetism, proton conduction and so on. Lanthanide ions are considered to be excellent metal ions for the construction of such systems because of their unique 4*f* electrons and can show remarkable photoluminescent, magnetic and catalytic properties. Among numerous ligands, pyridine­carboxyl­ate ligands bearing O and N coordination atoms have attracted considerable inter­est and have proved to be a class of excellent bridging linkers in fabricating metal coordination complexes with appealing structures. The 3-pyridyl­acetate ligand (3-PAA), one of the most simple pyridine­carboxyl­ate ligands, has attracted particular inter­est owing to its strong coordination and varied coordination modes, resulting in diverse structures with excellent properties. So far, coordination complexes constructed by the 3-PAA ligand have focused on transition-metal cations, but lanthanide complexes based on the 3-PAA ligand are still rare. Thus, in this work, we prepared the title compound [Dy_2_(*μ*_2_-PAA)_4_(Cl)_2_(phen)_2_] (**1**) (3-PAA = 3-pyridyl­acetate, phen = 1,10-phenanthroline), which displays a dinuclear structure.

**Figure 1:**
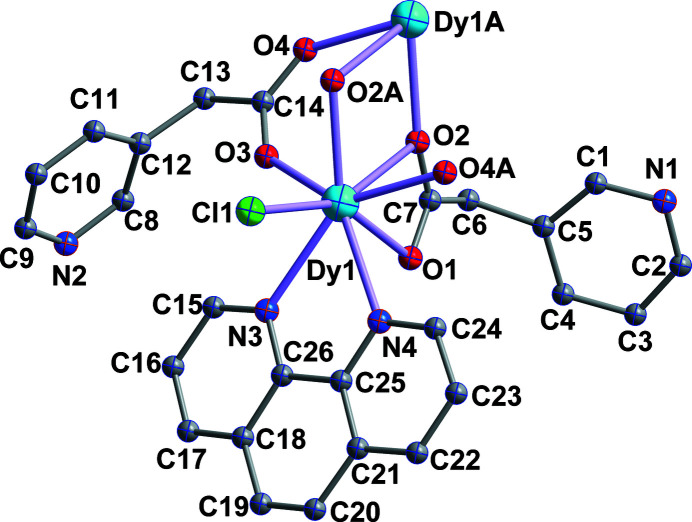
The asymmetric unit of **1** with 40% probability displacement ellipsoids. H atoms are omitted for clarity. Symmetry code: (A) 1 − *x*, 1 − *y*, 2 − *z*.

**Figure 2:**
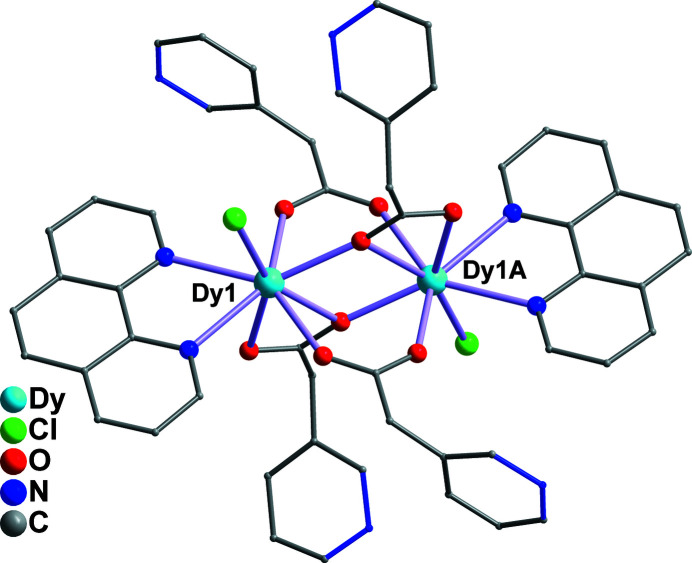
The dinuclear structure of **1**. H atoms are omitted for clarity. Symmetry code: (A) 1 − *x*, 1 − *y*, 2 − *z*.

**Figure 3:**
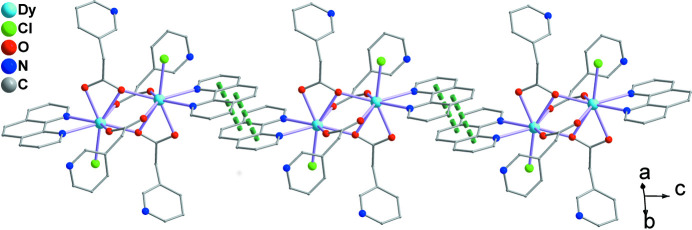
The supra­molecular chain along the *c*-axis direction formed by face-to-face *π*–*π* stacking inter­actions.

The asymmetric unit of **1** (Fig. 1 ▸) consists of one crystallographically independent Dy^III^ ion, one Cl^−^ anion, two PAA ligands and one phen mol­ecule. The Dy^III^ cation is eight-coordinated by five carboxyl­ate oxygen atoms from four different PAA^−^ ligands, one Cl^−^ ion, and two nitro­gen atoms from one chelating phen mol­ecule. The Dy—O bond lengths range from 2.3069 (17) to 2.5170 (15) Å, and the Dy—N bond distances are 2.5386 (18) and 2.5516 (17)Å, which are similar to those in the complex [Zn(*μ*-*L*)(*μ*-dicl)Dy(NO_3_)_2_]·H_2_O {*L* = *N*,*N*′-dimethyl-*N*,*N*′-bis­(2-hy­droxy-3-formyl-5-bromo­benzyl, dicl = deprotonated diclofenac = 2-[(2,6-di­chloro­phen­yl)amino] benzene acetate; Echenique-Errandonea *et al.*, 2019 ▸}. The two PAA^−^ ligands exhibit two different coordination modes. One acts as a tridentate ligand with a *μ*_2_-η^1^:η^2^ mode, while the other serves as a bidentate ligand with a *μ*_2_-κ*O* ^3^:κ*O* ^4^ mode. It is worth emphasizing that the N atom of the PAA ligand is noncoordinating in **1**. As shown in Fig. 2 ▸, two neighboring Dy^III^ ions are linked by four bridging carboxyl groups of four PAA^−^ ligands, forming the binuclear structure of **1**, in which the nearest Dy⋯Dy separation is 3.8976 (19) Å. These adjacent binuclear dimers are further connected *via* face-to-face *π*–*π* stacking inter­actions involving the phenyl and pyridine rings of the phen ligands, the centroid-to-centroid distance being 3.6116 (10) Å, leading to the formation of supra­molecular chain**s** along the *c-*axis direction (Fig. 3 ▸). For background information on the lanthanide ions and the 3-pyridyl­acetic acid ligand, see: Chakraborty *et al.* (2021 ▸); Xin *et al.* (2019 ▸); Ma *et al.* (2020 ▸); Wang *et al.* (2011 ▸); Teo *et al.* (2009 ▸); Adams *et al.* (2006 ▸).

## Synthesis and crystallization

Dy(ClO_4_)_3_ (0.2 mmol), 3-pyridyl­acetic acid (3-PAA, 0.25 mmol), 1,10-phenanthroline (0.25 mmol), HCl (0.25mmol) and Et_3_N were dissolved in 5 mL of aceto­nitrile and then sealed into a 25 mL Teflon-lined stainless steel vessel. The vessel was kept at 433 K for 3 d under autogenous pressure and then cooled to room temperature at a rate of 5.63 K h^−1^. Colorless block-shaped crystals were obtained by filtration of the resulting solution. Yield based on Dy: 38%.

## Refinement

Crystal data, data collection and structure refinement details are summarized in Table 1 ▸. The instruction "delu 0.002 0.001 C11 C12" was used during the refinement to limit the displace­ment parameters of the specified atoms.

## Supplementary Material

Crystal structure: contains datablock(s) I, New_Global_Publ_Block. DOI: 10.1107/S2414314622002310/vm4049sup1.cif

CCDC reference: 2155088

Additional supporting information:  crystallographic information; 3D view; checkCIF report

## Figures and Tables

**Table 1:** Experimental details

Crystal data	
Chemical formula	[Dy_2_(C_7_H_6_NO_2_)_4_Cl_2_(C_12_H_8_N_2_)_2_]
*M*_r_	1300.82
Crystal system, space group	Monoclinic, *P*2_1_/*c*
Temperature (K)	296
*a*, *b*, *c* (Å)	8.8922 (1), 21.5425 (3), 12.9887 (1)
β (°)	101.755 (1)
*V* (Å^3^)	2435.94 (5)
*Z*	2
Radiation type	Mo *K*α
μ (mm^−1^)	3.22
Crystal size (mm)	0.22 × 0.20 × 0.19

Data collection	
Diffractometer	Bruker SAINT CCD area detector
Absorption correction	Multi-scan (*SADABS*; Bruker, 2008 ▸)
*T*_min_, *T*_max_	0.626, 0.746
No. of measured, independent and observed [*I* > 2σ(*I*)] reflections	16420, 4487, 3954
*R*_int_	0.024
(sin θ/λ)_max_ (Å^−1^)	0.606

Refinement	
*R*[*F* ^2^ > 2σ(*F* ^2^)], *wR*(*F* ^2^), *S*	0.021, 0.056, 1.01
No. of reflections	4487
No. of parameters	325
No. of restraints	1
H-atom treatment	H-atom parameters constrained
Δρ_max_, Δρ_min_ (e Å^−3^)	0.59, −0.34

Computer programs: *SMART* and *SAINT* (Bruker, 2008 ▸), *SHELXS97* (Sheldrick, 2008 ▸), and *SHELXTL* (Sheldrick, 2008 ▸).

## full crystallographic data

### Crystal data

**Table:**

[Dy_2_(C_7_H_6_NO_2_)_4_Cl_2_(C_12_H_8_N_2_)_2_]	*F*(000) = 1276
*M**_r_* = 1300.82	*D*_x_ = 1.773 Mg m^−^^3^
Monoclinic, *P*2_1_/*c*	Mo *K*α radiation, λ = 0.71073 Å
*a* = 8.8922 (1) Å	Cell parameters from 7687 reflections
*b* = 21.5425 (3) Å	θ = 2.5–27.3°
*c* = 12.9887 (1) Å	µ = 3.22 mm^−^^1^
β = 101.755 (1)°	*T* = 296 K
*V* = 2435.94 (5) Å^3^	Block, yellow
*Z* = 2	0.22 × 0.20 × 0.19 mm

### Data collection

**Table:**

Bruker SAINT CCD area detector diffractometer	4487 independent reflections
Radiation source: fine-focus sealed tube	3954 reflections with *I* > 2σ(*I*)
Graphite monochromator	*R*_int_ = 0.024
phi and ω scans	θ_max_ = 25.5°, θ_min_ = 1.9°
Absorption correction: multi-scan (SADABS; Bruker, 2008)	*h* = −10→10
*T*_min_ = 0.626, *T*_max_ = 0.746	*k* = −26→23
16420 measured reflections	*l* = −15→15

### Refinement

**Table:**

Refinement on *F*^2^	Primary atom site location: structure-invariant direct methods
Least-squares matrix: full	Secondary atom site location: difference Fourier map
*R*[*F*^2^ > 2σ(*F*^2^)] = 0.021	Hydrogen site location: inferred from neighbouring sites
*wR*(*F*^2^) = 0.056	H-atom parameters constrained
*S* = 1.00	*w* = 1/[σ^2^(*F*_o_^2^) + (0.035*P*)^2^] where *P* = (*F*_o_^2^ + 2*F*_c_^2^)/3
4487 reflections	(Δ/σ)_max_ = 0.002
325 parameters	Δρ_max_ = 0.59 e Å^−^^3^
1 restraint	Δρ_min_ = −0.34 e Å^−^^3^

### Special details

**Table:**

Geometry. All esds (except the esd in the dihedral angle between two l.s. planes) are estimated using the full covariance matrix. The cell esds are taken into account individually in the estimation of esds in distances, angles and torsion angles; correlations between esds in cell parameters are only used when they are defined by crystal symmetry. An approximate (isotropic) treatment of cell esds is used for estimating esds involving l.s. planes.
Refinement. Refinement of F^2^ against ALL reflections. The weighted R-factor wR and goodness of fit S are based on F^2^, conventional R-factors R are based on F, with F set to zero for negative F^2^. The threshold expression of F^2^ > 2sigma(F^2^) is used only for calculating R-factors(gt) etc. and is not relevant to the choice of reflections for refinement. R-factors based on F^2^ are statistically about twice as large as those based on F, and R- factors based on ALL data will be even larger. H atoms were placed in calculated positions with C—H = 0.93 Å in phenyl and pyridine rings while C–H = 0.97 Å in CH_2_ groups and refined in riding mode with *U*_iso_(H) = 1.2*U*_eq_(C).

### Fractional atomic coordinates and isotropic or equivalent isotropic displacement parameters (Å^2^)

**Table:**

	*x*	*y*	*z*	*U*_iso_*/*U*_eq_	
Dy1	0.387207 (12)	0.510416 (5)	0.855827 (7)	0.03127 (3)	
Cl1	0.13833 (7)	0.58009 (3)	0.81071 (5)	0.05938 (19)	
O3	0.57734 (18)	0.58550 (8)	0.90135 (12)	0.0487 (5)	
N3	0.4153 (2)	0.55350 (10)	0.67882 (13)	0.0420 (5)	
O1	0.57990 (18)	0.44419 (8)	0.80320 (11)	0.0472 (4)	
N4	0.2364 (2)	0.45183 (9)	0.69718 (13)	0.0383 (5)	
C7	0.6642 (3)	0.43988 (11)	0.89307 (17)	0.0365 (6)	
C14	0.6799 (3)	0.60069 (12)	0.97945 (18)	0.0455 (6)	
C18	0.3415 (3)	0.54877 (14)	0.48832 (18)	0.0544 (7)	
C26	0.3341 (3)	0.52662 (12)	0.58975 (17)	0.0416 (6)	
C25	0.2402 (3)	0.47369 (12)	0.59934 (17)	0.0420 (6)	
C21	0.1571 (3)	0.44537 (15)	0.50734 (18)	0.0545 (8)	
C6	0.8200 (3)	0.41057 (12)	0.90869 (18)	0.0441 (6)	
H6A	0.8755	0.4280	0.8585	0.053*	
H6B	0.8770	0.4202	0.9788	0.053*	
C24	0.1525 (3)	0.40185 (12)	0.7037 (2)	0.0498 (7)	
H24A	0.1480	0.3871	0.7702	0.060*	
C5	0.8108 (3)	0.34153 (13)	0.8952 (2)	0.0476 (7)	
C19	0.2538 (4)	0.51931 (16)	0.3983 (2)	0.0687 (10)	
H19A	0.2583	0.5344	0.3319	0.082*	
C22	0.0729 (3)	0.39253 (16)	0.5185 (2)	0.0696 (9)	
H22A	0.0181	0.3724	0.4592	0.084*	
C12	0.7310 (3)	0.69858 (12)	0.8813 (2)	0.0494 (7)	
C8	0.7993 (3)	0.70351 (14)	0.7954 (2)	0.0662 (9)	
H8A	0.8829	0.6781	0.7929	0.079*	
C11	0.6079 (3)	0.73727 (15)	0.8813 (2)	0.0690 (9)	
H11A	0.5574	0.7359	0.9374	0.083*	
C23	0.0701 (3)	0.36993 (15)	0.6167 (2)	0.0657 (9)	
H23A	0.0149	0.3343	0.6252	0.079*	
N2	0.7518 (3)	0.74312 (14)	0.7148 (2)	0.0894 (9)	
C17	0.4394 (4)	0.59840 (15)	0.4816 (2)	0.0658 (8)	
H17A	0.4493	0.6133	0.4161	0.079*	
C13	0.7883 (3)	0.65317 (14)	0.9671 (2)	0.0682 (9)	
H13A	0.8129	0.6757	1.0331	0.082*	
H13B	0.8831	0.6352	0.9547	0.082*	
O2	0.61601 (17)	0.46036 (8)	0.97223 (11)	0.0421 (4)	
O4	0.70410 (19)	0.57482 (8)	1.06850 (12)	0.0509 (5)	
C15	0.5041 (3)	0.60095 (13)	0.6677 (2)	0.0565 (8)	
H15A	0.5597	0.6197	0.7281	0.068*	
C20	0.1653 (4)	0.47066 (18)	0.4064 (2)	0.0720 (10)	
H20A	0.1078	0.4527	0.3458	0.086*	
C16	0.5196 (4)	0.62483 (14)	0.5697 (2)	0.0690 (9)	
H16A	0.5842	0.6583	0.5659	0.083*	
C1	0.7804 (4)	0.30426 (16)	0.9734 (3)	0.0882 (12)	
H1A	0.7679	0.3235	1.0352	0.106*	
C10	0.5589 (4)	0.77733 (16)	0.8009 (3)	0.0875 (12)	
H10A	0.4759	0.8035	0.8011	0.105*	
C4	0.8326 (4)	0.31262 (18)	0.8051 (3)	0.0958 (12)	
H4A	0.8526	0.3356	0.7487	0.115*	
N1	0.7671 (5)	0.24248 (16)	0.9688 (3)	0.1415 (16)	
C3	0.8239 (5)	0.2480 (2)	0.8001 (4)	0.1398 (18)	
H3A	0.8416	0.2267	0.7413	0.168*	
C9	0.6330 (5)	0.77806 (17)	0.7218 (3)	0.0939 (13)	
H9A	0.5980	0.8055	0.6668	0.113*	
C2	0.7889 (5)	0.2167 (2)	0.8833 (5)	0.140 (2)	
H2A	0.7800	0.1738	0.8781	0.168*	

### Atomic displacement parameters (Å^2^)

**Table:**

	*U*^11^	*U*^22^	*U*^33^	*U*^12^	*U*^13^	*U*^23^
Dy1	0.03380 (6)	0.03284 (7)	0.02427 (6)	0.00167 (4)	−0.00091 (4)	0.00130 (4)
Cl1	0.0556 (4)	0.0664 (4)	0.0473 (3)	0.0255 (3)	−0.0104 (3)	−0.0088 (3)
O3	0.0544 (10)	0.0502 (11)	0.0343 (8)	−0.0156 (8)	−0.0077 (7)	0.0103 (8)
N3	0.0500 (11)	0.0439 (12)	0.0303 (9)	0.0042 (10)	0.0043 (8)	0.0054 (8)
O1	0.0509 (9)	0.0605 (11)	0.0286 (8)	0.0160 (9)	0.0047 (7)	−0.0012 (7)
N4	0.0372 (10)	0.0439 (12)	0.0312 (9)	0.0055 (9)	0.0009 (8)	−0.0030 (8)
C7	0.0383 (12)	0.0338 (13)	0.0362 (12)	0.0016 (10)	0.0051 (10)	0.0017 (10)
C14	0.0493 (14)	0.0429 (15)	0.0403 (13)	−0.0087 (12)	−0.0003 (11)	0.0052 (11)
C18	0.0604 (16)	0.0681 (19)	0.0349 (13)	0.0217 (14)	0.0098 (11)	0.0097 (12)
C26	0.0482 (14)	0.0459 (15)	0.0290 (11)	0.0127 (12)	0.0041 (10)	0.0035 (10)
C25	0.0397 (13)	0.0538 (15)	0.0303 (12)	0.0140 (12)	0.0020 (10)	−0.0057 (11)
C21	0.0440 (14)	0.080 (2)	0.0353 (13)	0.0073 (14)	−0.0017 (11)	−0.0138 (13)
C6	0.0365 (12)	0.0509 (16)	0.0445 (13)	0.0049 (11)	0.0071 (10)	−0.0035 (11)
C24	0.0494 (14)	0.0535 (17)	0.0448 (14)	−0.0060 (13)	0.0053 (11)	−0.0079 (12)
C5	0.0319 (12)	0.0499 (16)	0.0566 (15)	0.0096 (11)	−0.0013 (11)	−0.0084 (12)
C19	0.078 (2)	0.095 (3)	0.0318 (14)	0.0236 (18)	0.0077 (14)	0.0053 (14)
C22	0.0583 (17)	0.097 (2)	0.0471 (15)	−0.0097 (18)	−0.0034 (13)	−0.0313 (16)
C12	0.0444 (13)	0.0429 (15)	0.0568 (15)	−0.0134 (10)	0.0005 (12)	0.0090 (12)
C8	0.0567 (17)	0.0577 (19)	0.086 (2)	−0.0059 (15)	0.0176 (16)	0.0155 (16)
C11	0.0628 (16)	0.067 (2)	0.078 (2)	−0.0037 (13)	0.0156 (16)	−0.0113 (17)
C23	0.0564 (16)	0.072 (2)	0.0648 (17)	−0.0175 (15)	0.0045 (14)	−0.0239 (15)
N2	0.101 (2)	0.090 (2)	0.0763 (17)	−0.0263 (18)	0.0171 (16)	0.0270 (16)
C17	0.0842 (19)	0.076 (2)	0.0398 (14)	0.0175 (18)	0.0187 (13)	0.0244 (14)
C13	0.0551 (16)	0.072 (2)	0.0658 (18)	−0.0288 (15)	−0.0150 (14)	0.0267 (15)
O2	0.0440 (9)	0.0520 (10)	0.0284 (8)	0.0135 (8)	0.0028 (7)	−0.0033 (7)
O4	0.0602 (11)	0.0502 (11)	0.0347 (8)	−0.0181 (9)	−0.0083 (8)	0.0070 (8)
C15	0.0703 (18)	0.0530 (17)	0.0455 (14)	−0.0066 (15)	0.0101 (13)	0.0103 (12)
C20	0.072 (2)	0.111 (3)	0.0272 (13)	0.016 (2)	−0.0040 (13)	−0.0135 (16)
C16	0.094 (2)	0.0609 (19)	0.0574 (17)	−0.0034 (17)	0.0266 (15)	0.0194 (14)
C1	0.128 (3)	0.057 (2)	0.065 (2)	−0.012 (2)	−0.016 (2)	0.0032 (17)
C10	0.069 (2)	0.051 (2)	0.129 (3)	0.0161 (17)	−0.011 (2)	0.004 (2)
C4	0.077 (2)	0.103 (3)	0.116 (3)	−0.003 (2)	0.041 (2)	−0.048 (2)
N1	0.183 (3)	0.057 (2)	0.146 (3)	−0.026 (2)	−0.057 (3)	0.026 (2)
C3	0.079 (2)	0.124 (3)	0.222 (4)	−0.001 (3)	0.044 (3)	−0.118 (3)
C9	0.103 (3)	0.060 (2)	0.100 (3)	−0.015 (2)	−0.025 (2)	0.026 (2)
C2	0.082 (3)	0.062 (3)	0.240 (6)	0.028 (2)	−0.052 (3)	−0.042 (3)

### Geometric parameters (Å, º)

**Table:**

Dy1—O4^i^	2.3069 (17)	C19—C20	1.327 (5)
Dy1—O3	2.3275 (16)	C19—H19A	0.9300
Dy1—O2^i^	2.3261 (14)	C22—C23	1.371 (4)
Dy1—O1	2.4323 (16)	C22—H22A	0.9300
Dy1—O2	2.5170 (15)	C12—C11	1.376 (4)
Dy1—N3	2.5386 (18)	C12—C8	1.378 (4)
Dy1—N4	2.5516 (17)	C12—C13	1.493 (4)
Dy1—Cl1	2.6392 (6)	C8—N2	1.350 (4)
Dy1—C7	2.850 (2)	C8—H8A	0.9300
Dy1—Dy1^i^	3.8976 (2)	C11—C10	1.356 (4)
O3—C14	1.261 (3)	C11—H11A	0.9300
N3—C15	1.317 (3)	C23—H23A	0.9300
N3—C26	1.362 (3)	N2—C9	1.315 (5)
O1—C7	1.256 (2)	C17—C16	1.346 (4)
N4—C24	1.323 (3)	C17—H17A	0.9300
N4—C25	1.362 (3)	C13—H13A	0.9700
C7—O2	1.271 (3)	C13—H13B	0.9700
C7—C6	1.498 (3)	O2—Dy1^i^	2.3261 (14)
C14—O4	1.262 (3)	O4—Dy1^i^	2.3069 (17)
C14—C13	1.515 (4)	C15—C16	1.405 (4)
C18—C19	1.417 (4)	C15—H15A	0.9300
C18—C26	1.415 (3)	C20—H20A	0.9300
C18—C17	1.393 (4)	C16—H16A	0.9300
C26—C25	1.434 (4)	C1—N1	1.336 (5)
C25—C21	1.409 (3)	C1—H1A	0.9300
C21—C22	1.386 (4)	C10—C9	1.329 (5)
C21—C20	1.435 (4)	C10—H10A	0.9300
C6—C5	1.498 (4)	C4—C3	1.395 (6)
C6—H6A	0.9700	C4—H4A	0.9300
C6—H6B	0.9700	N1—C2	1.291 (7)
C24—C23	1.396 (4)	C3—C2	1.362 (7)
C24—H24A	0.9300	C3—H3A	0.9300
C5—C1	1.364 (4)	C9—H9A	0.9300
C5—C4	1.374 (4)	C2—H2A	0.9300
			
O4^i^—Dy1—O3	138.19 (5)	C25—C21—C20	119.7 (3)
O4^i^—Dy1—O2^i^	74.46 (6)	C5—C6—C7	112.05 (19)
O3—Dy1—O2^i^	73.64 (6)	C5—C6—H6A	109.2
O4^i^—Dy1—O1	88.97 (6)	C7—C6—H6A	109.2
O3—Dy1—O1	87.84 (6)	C5—C6—H6B	109.2
O2^i^—Dy1—O1	125.15 (5)	C7—C6—H6B	109.2
O4^i^—Dy1—O2	73.42 (6)	H6A—C6—H6B	107.9
O3—Dy1—O2	71.86 (6)	N4—C24—C23	124.0 (3)
O2^i^—Dy1—O2	72.89 (6)	N4—C24—H24A	118.0
O1—Dy1—O2	52.27 (5)	C23—C24—H24A	118.0
O4^i^—Dy1—N3	141.73 (6)	C1—C5—C4	116.8 (3)
O3—Dy1—N3	77.03 (6)	C1—C5—C6	120.8 (3)
O2^i^—Dy1—N3	142.50 (7)	C4—C5—C6	122.4 (3)
O1—Dy1—N3	75.81 (6)	C20—C19—C18	121.6 (3)
O2—Dy1—N3	118.94 (6)	C20—C19—H19A	119.2
O4^i^—Dy1—N4	77.16 (6)	C18—C19—H19A	119.2
O3—Dy1—N4	141.62 (6)	C21—C22—C23	120.1 (3)
O2^i^—Dy1—N4	143.32 (6)	C21—C22—H22A	120.0
O1—Dy1—N4	76.54 (5)	C23—C22—H22A	120.0
O2—Dy1—N4	120.04 (6)	C11—C12—C8	115.8 (3)
N3—Dy1—N4	65.29 (6)	C11—C12—C13	123.2 (3)
O4^i^—Dy1—Cl1	101.26 (5)	C8—C12—C13	121.0 (3)
O3—Dy1—Cl1	101.19 (5)	N2—C8—C12	123.8 (3)
O2^i^—Dy1—Cl1	83.42 (4)	N2—C8—H8A	118.1
O1—Dy1—Cl1	151.41 (4)	C12—C8—H8A	118.1
O2—Dy1—Cl1	156.30 (4)	C12—C11—C10	121.0 (3)
N3—Dy1—Cl1	79.86 (5)	C12—C11—H11A	119.5
N4—Dy1—Cl1	79.81 (4)	C10—C11—H11A	119.5
O4^i^—Dy1—C7	82.55 (6)	C24—C23—C22	118.2 (3)
O3—Dy1—C7	76.68 (6)	C24—C23—H23A	120.9
O2^i^—Dy1—C7	99.24 (6)	C22—C23—H23A	120.9
O1—Dy1—C7	25.99 (5)	C9—N2—C8	115.9 (3)
O2—Dy1—C7	26.47 (5)	C16—C17—C18	120.1 (3)
N3—Dy1—C7	96.17 (6)	C16—C17—H17A	120.0
N4—Dy1—C7	99.62 (6)	C18—C17—H17A	120.0
Cl1—Dy1—C7	175.87 (5)	C12—C13—C14	116.1 (2)
O4^i^—Dy1—Dy1^i^	69.87 (4)	C12—C13—H13A	108.3
O3—Dy1—Dy1^i^	68.34 (4)	C14—C13—H13A	108.3
O2^i^—Dy1—Dy1^i^	38.11 (4)	C12—C13—H13B	108.3
O1—Dy1—Dy1^i^	87.04 (3)	C14—C13—H13B	108.3
O2—Dy1—Dy1^i^	34.78 (3)	H13A—C13—H13B	107.4
N3—Dy1—Dy1^i^	141.81 (4)	C7—O2—Dy1^i^	160.55 (14)
N4—Dy1—Dy1^i^	143.32 (4)	C7—O2—Dy1	91.55 (12)
Cl1—Dy1—Dy1^i^	121.533 (15)	Dy1^i^—O2—Dy1	107.11 (6)
C7—Dy1—Dy1^i^	61.17 (4)	C14—O4—Dy1^i^	137.13 (15)
C14—O3—Dy1	138.89 (15)	N3—C15—C16	123.7 (3)
C15—N3—C26	117.5 (2)	N3—C15—H15A	118.1
C15—N3—Dy1	123.72 (16)	C16—C15—H15A	118.1
C26—N3—Dy1	118.73 (16)	C19—C20—C21	120.9 (3)
C7—O1—Dy1	95.93 (14)	C19—C20—H20A	119.6
C24—N4—C25	117.5 (2)	C21—C20—H20A	119.6
C24—N4—Dy1	124.18 (15)	C17—C16—C15	118.9 (3)
C25—N4—Dy1	118.28 (15)	C17—C16—H16A	120.6
O1—C7—O2	119.4 (2)	C15—C16—H16A	120.6
O1—C7—C6	121.2 (2)	N1—C1—C5	125.6 (4)
O2—C7—C6	119.44 (19)	N1—C1—H1A	117.2
O1—C7—Dy1	58.08 (12)	C5—C1—H1A	117.2
O2—C7—Dy1	61.98 (11)	C9—C10—C11	118.1 (3)
C6—C7—Dy1	172.26 (17)	C9—C10—H10A	120.9
O4—C14—O3	125.6 (2)	C11—C10—H10A	120.9
O4—C14—C13	115.7 (2)	C5—C4—C3	118.4 (4)
O3—C14—C13	118.7 (2)	C5—C4—H4A	120.8
C19—C18—C26	119.7 (3)	C3—C4—H4A	120.8
C19—C18—C17	122.6 (3)	C2—N1—C1	116.0 (4)
C26—C18—C17	117.7 (2)	C2—C3—C4	118.4 (4)
N3—C26—C18	122.0 (2)	C2—C3—H3A	120.8
N3—C26—C25	118.8 (2)	C4—C3—H3A	120.8
C18—C26—C25	119.2 (2)	N2—C9—C10	125.3 (3)
N4—C25—C21	122.2 (2)	N2—C9—H9A	117.4
N4—C25—C26	118.8 (2)	C10—C9—H9A	117.4
C21—C25—C26	119.0 (2)	N1—C2—C3	124.7 (4)
C22—C21—C25	117.9 (2)	N1—C2—H2A	117.6
C22—C21—C20	122.4 (3)	C3—C2—H2A	117.6

Symmetry code: (i) −*x*+1, −*y*+1, −*z*+2.
